# The performance of plasma amyloid beta measurements in identifying amyloid plaques in Alzheimer’s disease: a literature review

**DOI:** 10.1186/s13195-022-01117-1

**Published:** 2022-12-27

**Authors:** Abby L. Brand, Paige E. Lawler, James G. Bollinger, Yan Li, Suzanne E. Schindler, Melody Li, Samir Lopez, Vitaliy Ovod, Akinori Nakamura, Leslie M. Shaw, Henrik Zetterberg, Oskar Hansson, Randall J. Bateman

**Affiliations:** 1 Department of Neurology, Washington University School of Medicine, St. Louis, MO USA; 2 The Tracy Family SILQ Center, Washington University School of Medicine, St. Louis, MO USA; 3 Knight Alzheimer’s Disease Research Center, Washington University School of Medicine, St. Louis, MO USA; 4 Department of Biomarker Research, National Center for Geriatrics and Gerontology, Obu, Japan; 5 Department of Cognition and Behavior Science, Nagoya University Graduate School of Medicine, Nagoya, Japan; 6 Department of Pathology and Laboratory Medicine, Perelman School of Medicine, University of Pennsylvania, Philadelphia, PA USA; 7 Department of Psychiatry and Neurochemistry, Institute of Neuroscience and Physiology, the Sahlgrenska Academy at the University of Gothenburg, Mölndal, Sweden; 8 Clinical Neurochemistry Laboratory, Sahlgrenska University Hospital, Mölndal, Sweden; 9 Department of Neurodegenerative Disease, UCL Institute of Neurology, Queen Square, London, UK; 10 UK Dementia Research Institute at UCL, London, UK; 11 Hong Kong Center for Neurodegenerative Diseases, Clear Water Bay, Hong Kong, China; 12 Clinical Memory Research Unit, Department of Clinical Sciences, Lund University, Malmö, Sweden; 13 Memory Clinic, Skåne University Hospital, Lund, Sweden; 14 Hope Center for Neurological Disorders, Washington University School of Medicine, St. Louis, MO USA

**Keywords:** Alzheimer’s disease, Biomarker, Blood, Plasma, Amyloid beta, Amyloidosis

## Abstract

The extracellular buildup of amyloid beta (Aβ) plaques in the brain is a hallmark of Alzheimer’s disease (AD). Detection of Aβ pathology is essential for AD diagnosis and for identifying and recruiting research participants for clinical trials evaluating disease-modifying therapies. Currently, AD diagnoses are usually made by clinical assessments, although detection of AD pathology with positron emission tomography (PET) scans or cerebrospinal fluid (CSF) analysis can be used by specialty clinics. These measures of Aβ aggregation, e.g. plaques, protofibrils, and oligomers, are medically invasive and often only available at specialized medical centers or not covered by medical insurance, and PET scans are costly. Therefore, a major goal in recent years has been to identify blood-based biomarkers that can accurately detect AD pathology with cost-effective, minimally invasive procedures.

To assess the performance of plasma Aβ assays in predicting amyloid burden in the central nervous system (CNS), this review compares twenty-one different manuscripts that used measurements of 42 and 40 amino acid-long Aβ (Aβ42 and Aβ40) in plasma to predict CNS amyloid status. Methodologies that quantitate Aβ42 and 40 peptides in blood via immunoassay or immunoprecipitation-mass spectrometry (IP-MS) were considered, and their ability to distinguish participants with amyloidosis compared to amyloid PET and CSF Aβ measures as reference standards was evaluated. Recent studies indicate that some IP-MS assays perform well in accurately and precisely measuring Aβ and detecting brain amyloid aggregates.

**Supplementary Information:**

The online version contains supplementary material available at 10.1186/s13195-022-01117-1.

## Background

Diagnoses for Alzheimer’s disease are assisted with the detection of pathology by measures of amyloid beta (Aβ) aggregates. These measures are often obtained through brain scans or collection of spinal fluid with lumbar punctures, which are not readily accessible to a large portion of the population. To combat this, researchers have studied technologies to measure Aβ in the blood yet have encountered long-standing challenges in accuracy, sensitivity, and specificity of these measures. By searching the literature for plasma Aβ biomarker studies with appropriate sample sizes and analyses from 2014 to 2022, this review aims to assess the current technologies that measure blood plasma Aβ and compare their clinical utilities for identifying amyloid plaques.

## Main text

### Introduction

The amyloid beta (Aβ) protein is a naturally occurring protein in the body formed from the proteolytic cleavage of the amyloid precursor protein. In Alzheimer’s disease (AD), abnormal levels of Aβ aggregate to form plaques in the brain which disrupt neuronal function. An increased level of Aβ aggregates in the brain is associated with increased progression of AD pathology and rates of cognitive decline [1]. The current standards for AD diagnosis are amyloid positron emission tomography (PET) imaging and cerebrospinal fluid (CSF) measurements of Aβ, sometimes used in combination with measurements of CSF tau forms [2]. However, these standards are medically invasive, require specially trained staff, and PET scans in particular are costly with low accessibility. This ultimately limits the application of these standards in a broad range of clinical care settings. Therefore, a reliable blood plasma-based biomarker for AD is critical for widespread clinical diagnosis and screening for clinical studies to investigate the effects of disease-modifying therapies, non-drug interventions, risk management, and lifestyles on AD progression [3–5].

**Fig. 1:**
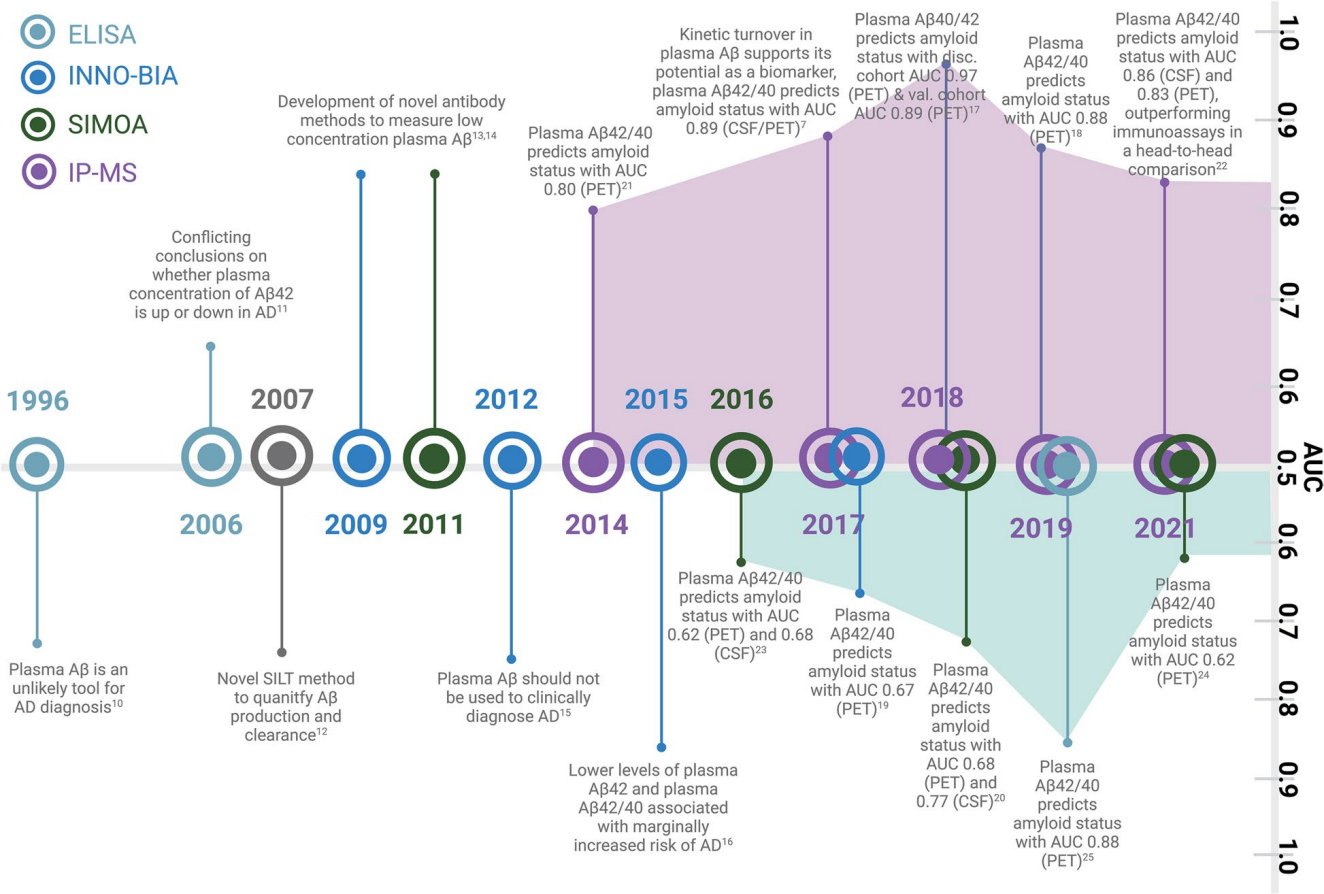
Timeline of Aβ studies [7, 10–25]. Timeline denoting significant events surrounding plasma Aβ use as a biomarker in AD diagnosis, color-coded by assay type. Results were conflicting for many years, but recent IP-MS studies provide promising AUC values for plasma Aβ42/40 measures. The diagnostic reference standard used in each study is listed in parentheses. For studies that used PET as a reference, the tracers include Pittsburg Compound B [7, 17–21, 25], flutemetamol [17, 20, 22, 23], florbetapir [17, 18, 20, 22, 24], and florbetaben [20]. Abbreviations: Disc., Discovery; Val., Validation. Figure created with BioRender.com

There have been long-standing challenges to obtaining accurate plasma Aβ measurements because concentrations of Aβ are 50–100 times lower in the plasma than in CSF [6]. In addition, there is a difference of less than 20% between plasma Aβ42/40 ratios in the disease state versus the non-disease state, compared with a 50% difference in CSF [2, 7, 8]. With prior high assay variability, it was difficult to determine group differences in AD vs. non-AD plasma Aβ due to the assays’ lack of sufficient precision. Consequently, studies of plasma Aβ as a biomarker for AD produced conflicting results and its utility was widely questioned for many years [9]. However, recent technological advancements in mass spectrometry have led to improvements in instrument sensitivity and precision which can detect femtomolar concentrations of protein with a coefficient of variation of less than 4%, resulting in the development of improved plasma Aβ assays. In the past few years, many studies reported encouraging results for plasma Aβ use as a biomarker for AD (Fig. 1). This review of twenty-one manuscripts evaluates the current potential of plasma Aβ as a diagnostic tool for AD.

### Methods

Studies were initially selected from Ashford et al. which included 73 articles in its systematic review of predictors of brain amyloid status [1]. This review was chosen due to its extensive search for studies on cost-effective methods to predict brain amyloid, all of which underwent a quality assessment. Ashford et al. categorized studies by their predictor, namely magnetic resonance imaging (MRI), cognitive measures, apolipoprotein E (*APOE*) genotype, plasma proteins, plasma amyloid, and various combined measures. Every study that used plasma amyloid as a predictor was evaluated as a candidate for the current review, and those that did not include receiver operating characteristic (ROC) analyses for the plasma Aβ42/40 ratio alone (in the absence of other factors such as age and *APOE* genotype) were excluded, narrowing the collection to eight manuscripts. ROC analyses are a useful tool for evaluating diagnostic tests, with the area under the ROC curve (AUC) as a summary of the test’s diagnostic accuracy. An AUC of 0.5 is equivalent to a test of random chance, while an AUC of 1.0 yields perfect diagnostic accuracy against a standard [26, 27].

Additional literature research was performed to ensure the inclusion of recent studies measuring plasma Aβ. Using a date range of 2014 to 2022 and keywords including plasma amyloid beta biomarker and amyloidosis, studies with plasma Aβ42/40 as the primary analysis with performance characteristics compared to PET or CSF with ROC analysis on a sufficient number of samples (greater than 50) were added to the review. Since age and *APOE* genotype alone provide a discriminative accuracy of about 0.75 between amyloid-positive and -negative individuals [28], only studies that found an AUC greater than 0.75 by plasma Aβ42/40 biomarker alone in at least one cohort were considered for this review. Following the additional literature search, four manuscripts with a head-to-head comparison of multiple assays, six IP-MS manuscripts, two high-sensitivity chemiluminescence enzyme immunoassay (ECL) manuscripts, and one single molecule array (SIMOA) manuscript were added for a total of twenty-one manuscripts in this review (see Additional file 1 for list of identified manuscripts as well as a schematic of the manuscript compilation strategy).

Each study was evaluated based on the characteristics of its cohort and the type of reference standard used, CSF Aβ or amyloid PET, which groups participants into positive or negative amyloid status as the ground truth. Parameters for evaluating the performance of plasma Aβ42/40, including the AUC, sensitivity, and specificity, were summarized.

### Results

**Fig. 2:**
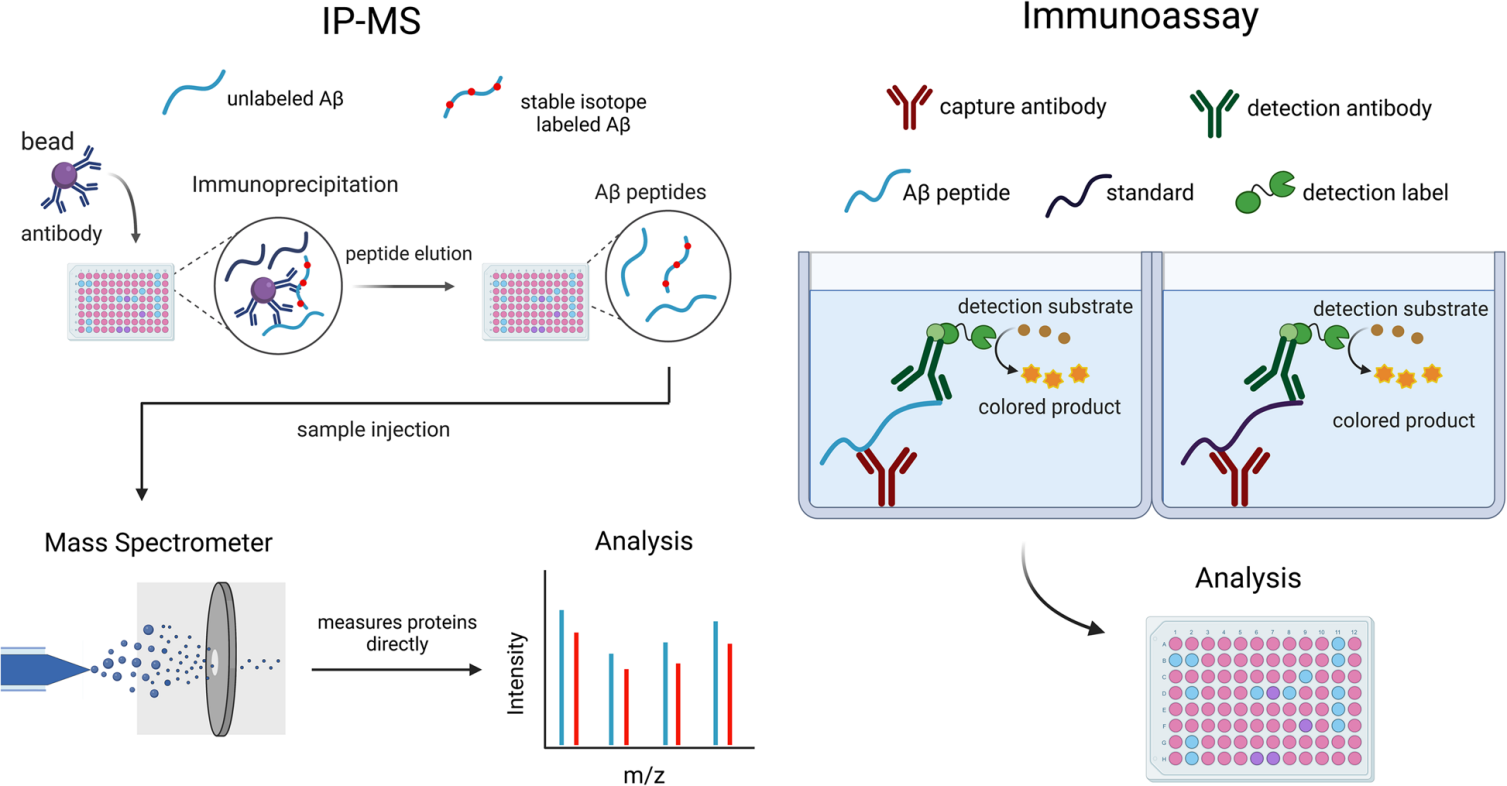
Contrasting methods to measure plasma Aβ. Two common methods to measure plasma Aβ are IP-MS assays (left) and immunoassays (right). In IP-MS assays, the detector measures Aβ species directly and quantitation is performed with an internal standard of stable isotope-labeled Aβ. In immunoassays, Aβ species are measured indirectly with antibody binding, and a different detection antibody must be used for each Aβ isoform. Immunoassays perform quantitation with an external standard. The immunoassay depicted in this figure is a plate-based sandwich immunoassay; bead-based immunoassays are also common, using fluorescently barcoded beads bound to an antibody for indirect measuring of a target. Figure created with BioRender.com

Many studies included in this review utilize high-precision IP-MS techniques in which Aβ species are first purified using antibody beads and then are directly measured in parallel by mass spectrometry so that Aβ42, Aβ40, and other species are measured together [7, 17, 18, 21, 22, 24, 28–33]. A similar technique applied by some studies is known as immunoprecipitation-free liquid chromatography-mass spectrometry (IP-free LC-MS), which measures Aβ species with mass spectrometry, but without antibody purification prior to measurement by LC-MS [22, 34]. Studies that use a bead-based immunoassay, for example, the SIMOA assay or some high-sensitivity chemiluminescence assays, use beads for specific Aβ species antibody binding and indirect quantification, sometimes after amplification [20, 22, 28, 35–40]. In contrast, other studies apply plate-based immunoassays (such as an ELISA assay), in which a binding antibody is adsorbed onto a plate where it binds the Aβ species, and a second antibody binds to another Aβ antigen, forming what is known as a “sandwich” between the two antibodies [22, 25, 28, 36]. The Aβ species is indirectly measured with an enzyme that generates a color signal, for colorimetric assays, or light, for chemiluminescence assays, proportional to the amount of antibody binding present in the sample (Fig. 2). One key component of this review is recognizing the additional error introduced into the plasma Aβ42/40 ratio with immunoassay techniques, as they measure plasma Aβ42 and plasma Aβ40 peptides separately, while IP-MS methods measure both simultaneously. Though immunoassays have been commonly used due to existing equipment, ease-of-use, and throughput, the most precise methods for diagnosis are especially important since the plasma Aβ42/40 ratio differs by less than 20% between the disease state and the non-disease state [2, 7, 8].

**Fig. 3:**
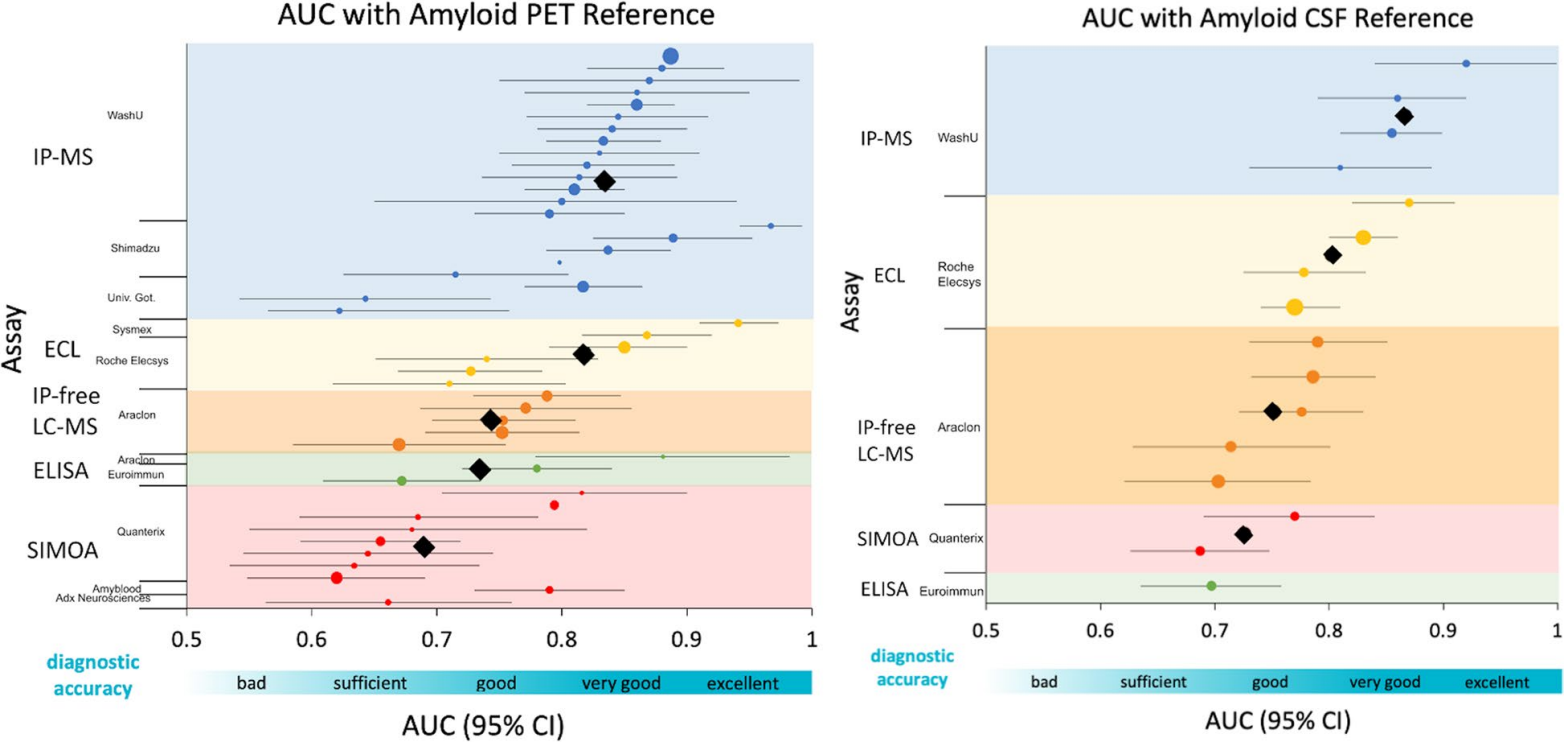
Forest plots of all AUC values with PET and CSF references. The points are categorized and color-coded by assay type, and the horizontal bars represent a 95% confidence interval. Blue is IP-MS assay, yellow is ECL, orange is an antibody-free LC-MS assay, green is ELISA, and red is SIMOA. The black diamond symbols represent the weighted average of the assays for each category, and within categories, the assay name is listed on the y-axis. The size of each point corresponds to the sample size of the cohort and the diagnostic accuracy of AUC values is depicted on a scale below the x-axis [27]. Abbreviations: WashU, Washington University; Univ. Got., University of Gothenburg

Of the six manuscripts that used CSF Aβ as the reference standard for amyloid status, all studies utilized the CSF Aβ42/40 ratio as the standard except for the Verberk et al. study, which used CSF Aβ42 levels. In a head-to-head comparison of five different assays on one cohort, the Washington University (WashU)-developed IP-MS assay outperformed all other assays with an AUC of 0.86 (95% CI 0.81–0.90) [22]. The IP-free LC-MS assay in this study had an AUC of 0.78 (95% CI 0.72–0.83), the bead-based SIMOA immunoassay had an AUC of 0.69 (95% CI 0.63–0.75), and the chemiluminescence and ELISA assays had AUCs of 0.78 (95% CI 0.73–0.83) and 0.70 (95% CI 0.64–0.76) respectively [22]. For all studies that used CSF as the reference standard, the weighted average of AUC values for IP-MS assays was 0.866 across four cohorts [22, 30, 31]. The weighted average AUC for chemiluminescence assays was 0.803 across four cohorts [26, 39, 40] and the weighted average AUC for SIMOA assays was 0.726 across two cohorts [20, 22]. The IP-free LC-MS assays had a weighted average AUC of 0.752 across five cohorts [22, 34] (Table 1, Fig. 3).

**Table 1:** Study data

Assay type	Study	Clinical study	Cohort size (average age)	Distribution of CN/MCI/AD	Amyloid positive rate	AUC (95% CI) of plasma Aβ42/40 in classifying amyloid PET status	PET tracer (amyloid status cut-off)	AUC (95% CI) of plasma Aβ42/40 in classifying amyloid CSF status	CSF amyloid status cut-off	Correlation	Sensitivity	Specificity	Plasma status cut-off as plasma Aβ42/40 ratio
IP-MS	Nakamura et al. 2018 [17] (Discovery cohort)	National Center for Geriatrics and Gerontology (NCGG)	*n*=121 (74.0 years)	62/30/29	41.3% by PET	0.967 (0.942–0.992) ^a^	PiB (1.4)	-	-	For PET, Pearson’s *r* ^a^= 0.767	0.960	0.873	3.93% ^a^
	(Validation cohort)	Australian Imaging, Biomarkers and Lifestyle Flagship Study of Aging (AIBL)	*n*=252 (74.2 years)	156/67/29	54.3% by PET	PiB: 0.889 (0.825–0.952) ^a^ Overall: 0.837 (0.787–0.887) ^a^	PiB (1.40), FMM (0.55), Florbetapir (1.05)	-	-	For PET, Pearson’s *r* ^a^= PiB: 0.601 FMM: 0.540 Florbetapir: 0.466	PiB: 0.733 Overall: 0.657	PiB: 0.922 Overall: 0.896	3.62% ^a^
ECL	Yamashita et al. 2022 [38] (Discovery cohort)	MissionAD	*n*=197 (71.1 years)	MCI due to AD/mild AD 157/40	50.8% by PET	0.941 (0.910–0.973)	FMM, Florbetapir, Florbetaben (based on visual read)	-	-	For PET, Spearman’s *r* ^b^= −0.75	0.96 (0.90–0.99)	0.84 (0.75–0.90)	10.2%
	(Validation cohort)	MissionAD	*n*=200 (70.8 years)	MCI due to AD/mild AD 160/40	50.0% by PET	0.868 (0.816–0.920)	FMM, Florbetapir, Florbetaben (based on visual read)	-	-	For PET, Spearman’s *r* ^b^= −0.73	0.88 (0.80–0.94)	0.72 (0.62–0.81)	10.2%
IP-MS	Ovod et al. 2017 [7]	Knight Alzheimer’s Disease Research Center (ADRC)	*n*=41 (76.2 years)	27/14 ^c^	43.9% by PET/CSF ^c^	0.887	PiB	-	-	For CSF, *r*=0.699	-	-	12.43%
ELISA	Pérez-Grijalba et al. 2019 [25]	AB255 Study	*n*=59 (72.7 years)	39/20/0	30.5% by PET	0.881 (0.779–0.982)	PiB (1.4)	-	-	For PET, Spearman’s *r*= −0.464	0.778	0.875	10.49%
IP-MS	Schindler et al. 2019 [18]	Clinical study from Washington University in St. Louis	*n*=158 (63.7 years)	CDR 0/0.5/1 ^d^: 148/9/1	27.2% by PET	0.88 (0.82–0.93)	PiB (1.42), Florbetapir (1.22)	-	-	For PET, Spearman’s *r*= −0.55 (−0.65 to −0.43)	0.88 (0.75–0.96)	0.76 (0.67–0.83)	12.18%
ECL	Palmqvist et al. 2022 [40]	Panel A+	*n*=227 (66.5 years)	32/106/89 ^e^	48.5% by CSF	-	-	0.87 (0.82–0.91)	0.047 ^f^	For CSF, Spearman’s *r*= 0.64	-	-	-
		BioFINDER-1	*n*=693 (72.4 years)^g^	461/232/0 (174 of CU participants had SCD)	41.8% by CSF	0.85 (0.79–0.90)	FMM (1.42)	0.83 (0.80–0.86)	0.066 ^f^	For CSF, Spearman’s *r*= 0.40	-	-	-
IP-MS	Hu et al. 2022 [33]	MissionAD	*n*=437 (72.4 years)	0/411/17 ^e^ (9 with no criteria met)	49.7% by PET	0.86 (0.82–0.89)	Florbetapir, Florbetaben (Centiloid>25)	-	-	-	0.90 (0.86–0.94)	0.71 (0.65–0.77)	9.2%
IP-MS	Schindler et al. 2022 [30]	Knight ADRC	*n*=152 (68.4 years) ^h^	CDR 0/0.5/1 ^d^: 138/9/5	32.5% by CSF	-	-	0.86 (0.79–0.92)	0.0673 ^f^	For CSF, Spearman’s *r*= 0.61 (0.50–0.70)	0.86	0.73	9.95%
		Knight ADRC	*n*=103 (68.4 years) ^h^	CDR 0/0.5/1 ^d^: 99/3/1	25.2% by PET	0.86 (0.77–0.95)	PiB (1.42), Florbetapir (1.19)	-	-	For PET, Spearman’s *r*= −0.44 (−0.58 to −0.27)	0.85	0.71	9.85%
Multiple	Janelidze et al. 2021 [22] (Validation cohort)	Alzheimer’s Disease Neuroimaging Initiative (ADNI)	*n*=122 (72.4 years)	51/51/20	48.3% by PET	IP-MS: 0.845 (0.772–0.917) ECL: 0.740 (0.651–0.829) SIMOA: 0.685 (0.590–0.781) IP-MS: 0.662 (0.565–0.758) SIMOA: 0.634 (0.534–0.734)	Florbetapir (1.11)	-	-	-	-	-	-
	(Development cohort)	Biomarkers For Identifying Neurodegenerative Disorders Early and Reliably (BioFINDER)	*n*=286 (71.6 years)	182/104/0	41.2% by CSF 38.4% by PET	IP-MS: 0.833 (0.787–0.879) IP-free LC-MS: 0.753 (0.696–0.811) ECL: 0.727 (0.669–0.784) ELISA: 0.672 (0.609–0.735) SIMOA: 0.655 (0.591–0.719)	FMM (1.42)	IP-MS: 0.855 (0.810–0.899) ECL: 0.778 (0.725–0.832) IP-free LC-MS: 0.776 (0.721–0.830) ELISA: 0.697 (0.635–0.758) SIMOA: 0.687 (0.626-0.748)	0.059 ^i^	For CSF, Spearman’s *r* ^b^= IP-MS: 0.66 ECL: 0.48 ELISA: 0.36 SIMOA: 0.31 IP-free LC-MS: 0.46	-	-	-
IP-MS	Li et al. 2022 [31]	AIBL	*n*=183 (74.2 years)	71/66/46	57.4% by PET	0.84 (0.78–0.90)	PiB, FMM, Florbetapir (Centiloid>25)	-	-	-	0.90 (0.82–0.95)	0.69 (0.58–0.79)	12.3%
		BioFINDER	*n*=100 (71.1 years)	28/51/0 (SMC or SCD=21)	50.0% by PET	0.83 (0.75–0.91)	FMM (1.42)	0.81 (0.73–0.89)	0.066 ^i^	For CSF, Spearman’s *r*= 0.59 (0.45–0.71) For PET, Spearman’s *r* = −0.52 (−0.65 to −0.35)	0.74 (0.60–0.85)	0.78 (0.64–0.88)	12.3%
		ADNI	*n*=182 (72.5 years)	71/86/0 (SMC or SCD=25)	48.9% by PET	0.82 (0.76–0.89)	Florbetapir (1.11)	0.92 (0.84–1)	0.064 ^i^	For CSF, Spearman’s *r* = 0.60 (0.31–0.79) For PET, Spearman’s *r*= −0.57 (−0.66 to −0.46)	0.73 (0.63–0.82)	0.84 (0.75–0.91)	12.5%
SIMOA	Tanaka et al. 2021 [37]	Recruited from the memory clinic at the National University Hospital, Singapore	*n*=68 (74.5 years)	14/0/15 (CIND=30, VaD=9)	33.8% by PET	0.816 (0.704–0.900)	PiB (visually interpreted)	-	-	For PET, Pearson’s *r*= 0.123	0.696	0.889	-
Multiple	Keshavan et al. 2021 [24]	Insight 46	*n*=441 (70.7 years)	410/7/0 (24 with prior neurological condition)	18.6% by PET	IP-MS: 0.817 (0.770–0.864) SIMOA: 0.62 (0.548–0.691)	Florbetapir (0.61)	-	-	-	IP-MS: 0.866 SIMOA: 0.451	IP-MS: 0.719 SIMOA: 0.780	IP-MS: 9.5% SIMOA: 5.8%
Multiple	Zicha et al. 2022 [28]	ADNI	*n*=121 (77.9 years)	49/54/18	49.6% by PET	IP-MS: 0.814 (0.736–0.892) 0.715 (0.625–0.805) ECL: 0.710 (0.617–0.803) SIMOA: 0.661 (0.563–0.760) 0.645 (0.545–0.745) IP-MS: 0.643 (0.542–0.743)	Florbetapir (1.11)	-	-	For PET, Spearman’s *r* ^j^ IP-MS: −0.533 −0.369 ECL: −0.390 SIMOA: −0.293 −0.329 IP-MS: −0.242	-	-	-
IP-MS	West et al. 2021 [32]	Clinical studies from University of Wisconsin, the Banner Alzheimer’s Institute, University of Florida, Washington University School of Medicine, and Precision for Medicine	*n*=414 (70.0 years) ^k^	-	39% by PET/CSF ^l^	0.81 (0.77–0.85)	PiB, Florbetapir, Florbetaben ^k^	-	-	-	-	-	9.75%
IP-MS	Tosun et al. 2021 [29]	ADNI	*n*=173 (72.5 years)	87/86/0	48.0% by PET	0.80 (0.65–0.94) ^m^ 0.87 (0.75–0.99) ^n^	Florbetapir (1.11)	-	-	-	0.64 ^m^ 0.79 ^n^	0.77 ^m^ 0.75 ^n^	-
IP-MS	Kaneko et al. 2014 [21]	NCGG	*n*=62 (74.1 years)	33/12/17	64.5% by PET	0.798	PiB (Scored by nuclear medicine physicians)	-	-	For PET, Pearson’s *r*= −0.316	0.750	0.773	
ECL	Palmqvist et al. 2019 [39] (Development cohort)	BioFINDER	*n*=842 (72 years)	513/265/64	43.7% by CSF	-	-	0.77 (0.74–0.81)	0.059 ^i^	For CSF, Spearman’s *r*= 0.476	0.75 (0.68–0.80)	0.72 (0.65–0.77)	6.5%
SIMOA	Vergallo et al. 2019 [35]	INSIGHT-preAD	*n*=276 (76.8 years)	276 with SMC	26.4% by PET	0.794 ^a^	Florbetapir (0.7918)	-	-	-	0.781	0.749	5.61% ^a^
IP-MS	Hu et al. 2022 [33]	Plasma Test for Amyloidosis Risk Screening (PARIS)	*n*=249 (74.6 years)	0/172/77	64.7% by PET	0.79 (0.73–0.85)	Florbetapir, Florbetaben, FMM (analyzed by radiologists)	-	-	-	0.85	0.63	8.9%
Multiple	De Meyer et al. 2020 [36]	Flemish Prevent AD Cohort KU Leuven (F-PACK) & Biomarker-based adaptive development in Alzheimer’s disease (BioAdaptAD)	*n*=199 (70 years)	161/38/0	19.1% by PET	SIMOA: 0.79 (0.73–0.85) ELISA: 0.78 (0.72–0.84)	FMM (1.38), Florbetaben (1.29)	-	-	For PET, Spearman’s *r*= SIMOA: −0.32 ELISA: −0.32 For CSF, Spearman’s *r* ^b,o^= SIMOA: 0.29 ELISA: 0.41	SIMOA: 0.74 (0.57–0.87) ELISA: 0.78 (0.62–0.90)	SIMOA: 0.80 (0.72–0.86) ELISA: 0.75 (0.68–0.82)	SIMOA: 23.0% ELISA: 15.9%
IP-free LC-MS	Janelidze et al. 2022 [34]	BioFINDER-1	*n*=381 (71.6 years) ^h, p^	241/140/0	43.1% by CSF 42.5% by PET	0.788 (0.729–0.847) ^m^ 0.771 (0.687–0.856) ^n^	FMM (0.74)	0.790 (0.730–0.851) ^m^ 0.714 (0.628–0.801) ^n^	0.091 ^q^	-	-	-	-
		BioFINDER-2	*n*=514 (66.2 years) ^h, r^	350/164/0	33.1% by CSF 31.7% by PET	0.752 (0.691–0.814) ^m^ 0.670 (0.585–0.755) ^n^	FMM (0.69)	0.786 (0.732–0.841) ^m^ 0.703 (0.621–0.784) ^n^	0.0752 ^s^	-	-	-	-
SIMOA	Verberk et al. 2018 [20]	Subjective Cognitive Impairment Cohort (SCIENCe) Project	*n*=248 (61.0 years)	248 with SCD	23% by CSF	-	-	0.77 (0.69–0.84)	CSF Aβ42 used ^t^: 813pg/mL	For CSF, Pearson’s *r*= 0.38	0.76	0.75	4.5%
		SCIENCe Project	*n*=69	69 with SCD	33% by PET	0.68 (0.55–0.82)	Florbetaben, Florbetapir, FMM, PiB (Scored by a nuclear medicine physician)	-	-	-	0.70	0.78	4.4%

*Abbreviations:* *PiB* Pittsburgh compound B, *FMM* [^18^F]flutemetamol, *CN* cognitively normal, *CU* cognitively unimpaired, *MCI* mild cognitive impairment, *CDR *Clinical Dementia Rating*,* *SMC* significant memory concern, *SCD* subjective cognitive decline, *CIND* cognitive impairment no dementia, *VaD* vascular dementia

^a^Study reports plasma Aβ40/42; converted cut-off to plasma Aβ42/40 for table

^b^Values for a subset of cohort

^c^*n*=27 CN (CDR=0), *n*=14 not CN (CDR>0). PET was used when available and CSF Aβ42 concentration otherwise

^d^CDR=0 is CN, CDR=0.5 is very mild dementia, and CDR=1 is mild dementia

^e^AD refers to mild AD

^f^Measured with a chemiluminescent enzyme immunoassay using a fully automated platform

^g^PET scans available for *n*=461, CSF measures available for all

^h^Median age is listed (not average)

^i^Measured by Elecsys immunoassays

^j^Correlation values for each assay are listed in the order corresponding to the AUC values

^k^Cohorts were from many different sites with no standardized criteria for clinical diagnoses. Each cohort had different PET SUVR cut-off values: 1.47 for PiB and florbetapir in cohort 3, 1.11 for florbetapir and 1.4 for florbetaben in cohort 5, and 1.42 for PiB and florbetapir in cohort 6 (PET status was binarized for cohort 4, cut-offs were not available for CSF determination of amyloid positivity). Values in the table are for the overall cohort

^l^For amyloid status, PET was used when available and CSF Aβ42/40 concentration otherwise, measured by ELISA or MS methods based on site

^m^For CU participants

^n^For MCI participants

^o^Correlation with CSF Aβ42/total-tau

^p^PET scans available for *n*=360, CSF measures available for all

^q^Measured by Euroimmun ELISA assays

^r^PET scans available for *n*=498, CSF measures available for all

^s^Measured by Meso Scale Discovery immunoassays

^t^Measured by Innotest ELISAs

The weighted average of AUC values for all studies that used an IP-MS assay with a PET reference is 0.834 across twenty-one cohorts [7, 17, 18, 21, 22, 24, 28–34]. The weighted average AUC for studies using the WashU-developed IP-MS assay with a PET standard is slightly higher, with a value of 0.846 across fourteen cohorts. In general, the immunoassays displayed lower AUCs across most studies that used a PET reference standard. Studies using a SIMOA assay had a weighted average AUC value of 0.690 across ten cohorts [20, 22, 24, 28, 35–37], chemiluminescence assays had a weighted average AUC of 0.818 across six cohorts [22, 28, 38, 40], IP-free LC-MS assays had a weighted average AUC of 0.742 across five cohorts [22, 34], and ELISA assays had a weighted average AUC of 0.734 across three cohorts [22, 25, 36] (Table 1, Fig. 3).

Within a head-to-head study of five different assays compared in the same cohort, the IP-MS assay outperformed all immunoassays against the PET standard, similar to findings when CSF Aβ was used as the reference standard [22]. In this study, the WashU IP-MS assay had an AUC of 0.83 (95% CI 0.79–0.88), the IP-free LC-MS assay had an AUC of 0.75 (95% CI 0.70–0.81), the SIMOA immunoassay had an AUC of 0.66 (95% CI 0.59–0.72), and the chemiluminescence and ELISA assays had an AUC of 0.73 (95% CI 0.67–0.78) and 0.67 (95% CI 0.61–0.74) respectively [22]. For the validation cohort of this study, two IP-MS assays had an average AUC of 0.755, and two SIMOA assays had an average AUC of 0.660 [22]. The chemiluminescence assay had an AUC of 0.74 (95% CI 0.65-0.83) [22]. A different head-to-head study employed a similar variety of assays on a cohort, with an average AUC of 0.723 for three IP-MS assays, while the WashU-developed assay alone had an AUC of 0.814 (95% CI 0.74-0.89) [28]. The chemiluminescence assay in this study had an AUC of 0.710 (95% CI 0.62-0.80) and two SIMOA assays had an average AUC of 0.655 [28] (Table 1, Fig. 3).

Due to the differences in cohorts between studies, no formal statistical analyses could be performed for this review. However, all studies reported AUC values that reflect the ability of each assay to predict amyloid status that is in agreement with the reference standard diagnosis.

### Discussion

Recent reviews of blood plasma tests broadly cover the various types of high-performance blood-based biomarkers that are utilized in research, including one that focused on mass spectrometry-based methods [8, 41–43]. These reviews have covered recent developments in amyloid, tau, neurodegeneration, and other biomarkers, but have not included in-depth reviews of blood Aβ measures, the relationships between Aβ assays, studies, and performance, and the implications for use in diagnostics and therapeutic programs. Because there are now clinically available blood tests for Aβ and an FDA-approved drug to remove amyloid plaques requiring clinical testing for amyloid, we chose to perform an extensive review comparing the different kinds of blood plasma Aβ42/40 ratio tests that have been developed.

In this review of plasma Aβ assays that assess Aβ42 and 40 values to predict amyloidosis, the IP-MS assays outperformed the immunoassays most times both in comparisons across studies and in the same cohort. One advantage to the IP-MS technique is the simultaneous quantification of the Aβ42 and Aβ40 peptides with an internal standard. This allows for only one opportunity for variance in the measurement, which is controlled by the internal standard, in contrast to the immunoassay methods which quantify each peptide separately and have independent errors associated with each because different antibodies must be used for each Aβ isoform with external standards [44]. In addition, the IP-MS method has superior analytical specificity to the immunoassays because the mass spectrometer measures Aβ species directly, while detection of Aβ is indirect with immunoassays (Fig. 2). Though immunoassays carry the benefit of being more widely used and somewhat less expensive, the diseased versus non-diseased plasma Aβ42/40 ratios differ by less than 20% in AD, so the most precise and accurate measure of the Aβ42/40 ratio is crucial to accurate diagnoses [2, 7, 8]. The enhanced precision and multiplexing capacity of the IP-MS methods have a definitive impact on the total error associated with the measurement of the two isoforms of Aβ that are used to derive the Aβ42/40 ratio. A recent study comparing IP-MS assays and immunoassays measuring plasma tau isoforms as a biomarker for AD has found that mass spectrometry-based tau phosphorylated at threonine 217 (p-tau217) performed significantly better than all plasma phosphorylated tau immunoassays when detecting abnormal Aβ status [45]. Higher precision and the fact that immunoassay antibody detection methods are more prone to blood plasma interferences are speculated as an explanation for why IP-MS assays have performed better than immunoassays in these studies.

With most assays, the plasma Aβ42/40 ratio had stronger predictive abilities when compared to the CSF Aβ standard than when compared to the PET Aβ standard. This is clearly illustrated in the Janelidze et al. (2021) study of five assays, the Verberk et al. study, the Janelidze et al. study (2022, BioFINDER-2 cohort), and the Li et al. study (ADNI cohort), all of which evaluated both standards in their respective cohorts (Table 1). This trend aligns with findings that CSF Aβ changes earlier in the disease process than amyloid PET, as well as findings that suggest plasma Aβ changes precede changes in amyloid PET [18, 46]. Exceptions to this trend include the Schindler et al. (2022) study, where the two standards performed equally, in addition to the Li et al. BioFINDER cohort, the Janelidze et al. 2022 BioFINDER-1 cohort (for the MCI group), and the Palmqvist et al. BioFINDER-1 cohort where the PET reference standard outperformed the CSF reference standard (Table 1). It is unclear why the CSF reference standard had a lower AUC than the PET reference in these groups, and the same assay showed better discriminative accuracy with the CSF reference standard on other cohorts included in these manuscripts. Additionally, different PET tracers correlate with plasma Aβ42/40 measures differently, and in future studies the PET tracer should be considered when interpreting results given that the percent of amyloid-positive individuals could account for variance between studies. In the Nakamura et al. study, PiB had higher AUC and correlation values with Aβ than other PET tracers (Table 1), consistent with findings that PiB is a more sensitive tracer than florbetapir [28, 47]. Considerations of the reference standards are important to note when evaluating AD biomarker studies, and independent comparisons of plasma Aβ, CSF Aβ, and amyloid PET should be made with pathology, clinical predictors, and response to treatment, as the most predictive measure is still not established.

Although using plasma Aβ as an AD biomarker was long questioned, recent studies have validated results for using plasma Aβ42/40 as a diagnostic tool for the detection of AD amyloid plaques. The weighted average of AUC values for all cohorts using an IP-MS assay in this review is 0.834 using PET as a reference standard and 0.866 using CSF as a reference standard. When diagnosing disease in patients, an AUC between 0.8 and 0.9 is considered very good [26, 27]. Even further, using plasma Aβ as a diagnostic tool for AD would confer significant benefits to the patient and healthcare community through decreased cost, invasiveness, and need for specially trained staff resulting in broader accessibility, diversity in research cohorts, and clinical access to diagnostic tests.

Mass spectrometry has been used in clinical labs for decades, and its use has expanded with commercial groups that can run millions of tests per year [48–51]. As automated and simplified clinical systems are available for sample processing and mass spectrometry analysis, specially trained staff are not required to run a developed clinical protocol and the ease of use approaches that of immunoassays [52–54]. Though the upfront cost of equipment for mass spectrometry assays is higher, the cost per sample is typically lower than that of immunoassays with similar materials (such as antibody, beads, enzyme, and solvent) and especially economical when screening for multiple analytes at one time [55, 56]. Therefore, the use of mass spectrometry assays on a wide scale is a practical choice for highly sensitive and accurate clinical blood tests.

Head-to-head comparisons similar to those described here enable statistical comparisons of assay performances that cannot be applied to studies utilizing different cohorts. Cross-sectional studies (AIBL, ADNI, NCGG, and BioFINDER) included in this review have compared Aβ assays in the same cohort; replicating their findings across cohorts is necessary for a robust conclusion on how assays compare. A challenge with plasma Aβ as a biomarker for cerebral Aβ pathology is the relatively small fold change between amyloid-positive and -negative individuals. This mandates a strong quality control system to avoid minor (less than 4%) longitudinal drift in the measurements. This challenge has been met with stable measures utilizing IP-MS in both the research and clinical setting demonstrating consistent differentiation between amyloid-positive and amyloid-negative across cohorts and years. Longitudinal studies of plasma Aβ measures should also be prioritized to confirm plasma Aβ predictability.

Assays should be tested in cohorts that are similar to the population expected to use the test. For almost every cohort evaluated in this review, a self-identified race was not reported. However, most AD research cohorts are comprised of individuals who identify as non-Hispanic White with high socioeconomic status. The assays should be tested in cohorts that are more representative of the general population to ensure accurate and consistent performance across groups, as AD research studies typically consist of volunteers with a high prevalence of family history of AD, high socioeconomic status, and limited co-morbidities. CSF and PET Aβ have been examined in various racial groups and studies have found inconsistent results regarding the relationship between amyloid biomarkers and race, possibly due to differences in recruitment, comorbidities, or other factors [30]. However, one study found that plasma Aβ42/40 performed consistently in the prediction of CSF and PET Aβ status across racial groups [30], and another found consistent results in Japanese and Australian populations [17]. Recent findings suggest each kind of biomarker should be evaluated for factors which influence it. For example, kidney disease has been shown to alter the plasma levels of neurofilament light chain (NfL), glial fibrillary acidic protein (GFAP), tau phosphorylated at threonine 181 (p-tau181), p-tau217, Aβ42 and Aβ40 measures, but the Aβ42/Aβ40 ratio is unaffected and the clinical performance of all the plasma markers does not seem to be significantly affected [57–60]. The reason for plasma Aβ42/Aβ40 ratio resilience to co-morbidity effects could be due to impacts on Aβ concentrations canceling out between the similar 42 and 40 amino acid sequences [58], also potentially the use of other amyloid species (e.g. amyloid precursor protein at amino acids 669-711, known as APP669-711) could be used [17, 21, 61].

It is important to consider the standard for the blood test may vary with context: in research and clinical trials, CSF and PET Aβ are the reference standards, whereas when used in the clinic for diagnosis, the clinical accuracy is the standard for comparison. Though PET, CSF, and blood biomarkers are not used as the sole means of an AD diagnosis, they are essential in determining which patients likely do or do not have AD amyloid plaques, and thus are expected to benefit from disease-modifying drugs. Therefore, the use of these biomarkers optimizes the inclusion of subjects in clinical trials [41]. Current estimates are that primary care clinics, which provide the majority of dementia care, are only 40–60% accurate in diagnosing AD due to underdiagnoses and misdiagnoses [2]. Having an accurate measure of AD pathology with a blood biomarker would improve the ability of clinicians to accurately diagnose patients and may be required to start treatments that target amyloid plaques. Accurate blood biomarker assays will also assist in the recruitment of more diverse cohorts for clinical trials as a blood draw is less invasive, less expensive, and more accessible for patients than a lumbar puncture or PET scan.

Appropriate use guidelines for blood-based biomarkers will be helpful to guide the immense and potentially urgent need for accurate diagnosis of AD in the clinic [41]. There are currently two clinical tests available in the U.S.A., and there will likely be more available soon. Some groups have begun to develop guidelines on blood test use to ensure the accurate measurement and interpretation of biomarker results in subjects.

In addition to the emerging role of plasma Aβ as a blood biomarker for AD, plasma measurements of tau phosphorylated at threonine 231 (p-tau231), p-tau181, p-tau217, and potentially others have shown promise in diagnostic capacity [62–64]. Studies show that plasma levels of p-tau217 start to change at the same time as CSF levels of p-tau217 when amyloid plaques first appear by amyloid PET and precede tau-PET positivity by 15 to 20 years [65, 66]. In addition, it has been shown that anti-amyloid drugs have downstream effects on tau metabolism, so plasma p-tau217 could serve as a useful tool in monitoring pharmacodynamic effects on amyloid pathology from these treatments [67]. Other emerging blood biomarkers for AD include the possible use of GFAP and β-synuclein [68–70]. As different plasma measurements show potential for accurate diagnoses of AD, some groups have aimed to use them together. For example, a study showed combining APP669-711 with Aβ improves diagnostic performance [21]. Another study combining three plasma biomarkers into a composite biomarker of plasma p-tau217, plasma Aβ42/40, and plasma NfL showed improved performance in predicting amyloidosis over any of the three measures alone [67]. Many studies have also shown increased performance with the inclusion of *APOE* genotype in their biomarker [18, 20, 22, 25, 28–33, 39, 40].

There are several limitations in this review including the diverse group of assay performances, the range of cohorts studied that are not directly comparable, and different research groups and analytic approaches. Factors such as prevalence of amyloid plaques, clinical stage, age, *APOE* genotype, and others across cohorts may impact the results of the study. Differences in preanalytical variables, such as blood collection and processing methods, also complicate the comparison across cohorts. Despite these differences in cohorts, a consistent picture has emerged about the relationship between blood plasma Aβ and amyloid plaques which has been validated across many cohorts and labs. Future research should study cross-sectional and longitudinal plasma Aβ measures in predicting amyloidosis, clinical use, impact of screening on research studies and impact on clinical care, diagnosis, and management including potential drugs that could modify amyloid plaques.

### Conclusions

Based on this review of twenty-one manuscripts, the performance of some plasma Aβ42/40 measures in predicting amyloidosis promises to aid in the accurate diagnosis of AD versus non-AD causes of cognitive impairment. There are already clinically available blood plasma Aβ42/40 tests available based on IP-MS technologies for symptomatic patients. Current guidelines do not recommend predictive testing for asymptomatic patients yet, especially without treatment or prevention options to act on [41]. It has been shown that screening patients with plasma Aβ42/40 could reduce the number of amyloid PET scans required by approximately 49–64% [18, 20, 25, 37, 39]. In addition to the economic benefits to the patient and healthcare community, an accurate blood biomarker test enables wide-scale testing of more diverse populations. This could benefit the diagnosis of AD in a clinical setting, improving access to accurate diagnosis for marginalized populations and reducing the financial burden and health risk associated with current diagnostic procedures for patients. Further studies analyzing a combined biomarker with plasma Aβ42/40 and other measurements may confer even more accurate diagnoses from blood samples and is a valuable future investigation.

## Supplementary Information

**Additional file 1: eTable 1.** List of Identified Manuscripts to be Included in Review includes all citations, reference numbers, and types of assays studied for manuscripts included in the review. **eFigure 1.** Diagram of Literature Search is a schematic depicting how manuscripts were found for inclusion in this review, including a list of references at each step.

## Data Availability

Data sharing is not applicable to this article as no datasets were generated or analyzed during the current study.

## Abbreviations

AD: Alzheimer’s disease
Aβ: Amyloid beta
PET: Positron emission tomography
CSF: Cerebrospinal fluid
CNS: Central nervous system
*APOE*: Apolipoprotein E
MRI: Magnetic resonance imaging
ROC: Receiver operating characteristic
AUC: Area under the receiver operating characteristic curve
IP-MS: Immunoprecipitation-mass spectrometry
SIMOA: Single molecule array
ELISA: Enzyme-linked immunosorbent assay
LC-MS: Liquid chromatography-mass spectrometry
IP: Immunoprecipitation
IA: Immunoassay
ECL: Chemiluminescence immunoassay
Val.: Validation
Disc.: Discovery
PiB: Pittsburgh compound B
FMM: [^18^F]flutemetamol
CN: Cognitively normal
MCI: Mild cognitive impairment
AD: Alzheimer’s disease
SMC: Significant memory concern
SCD: Subjective cognitive decline
CIND: Cognitive impairment no dementia
VaD: Vascular dementia
BioFINDER: Biomarkers For Identifying Neurodegenerative Disorders Early and Reliably
ADRC: Alzheimer Disease Research Center
WashU: Washington University
Univ. Got.: University of Gothenburg
ADNI: Alzheimer’s Disease Neuroimaging Initiative, NCGG, National Center for Geriatrics and Gerontology
AIBL: Australian Imaging, Biomarkers and Lifestyle Study of Aging
SCIENCe: Subjective Cognitive Impairment Cohort
PARIS: Plasma Test for Amyloidosis Risk Screening
F-PACK: Flemish Prevent AD Cohort KU Leuven
BioAdaptAD: Biomarker-based adaptive development in Alzheimer’s disease
WashU: Washington University in St. Louis
NfL: Neurofilament light chain
p-tau217: Tau phosphorylated at threonine 217
p-tau181: Tau phosphorylated at threonine 181
p-tau231: Tau phosphorylated at threonine 231
APP669-711: Amyloid precursor protein at amino acids 669-711
