# Supplementary material for: The performance of plasma amyloid beta measurements in identifying amyloid plaques in Alzheimer’s disease: a literature review
Source: Alzheimers Res Ther. 2022 Dec 27;14:195. doi: 10.1186/s13195-022-01117-1 (PMC9793600; doi:10.1186/s13195-022-01117-1)

Additional File 1

eTable 1. List of Identified Manuscripts to be Included in Review

| Assay Type | Reference # | Manuscript |
| --- | --- | --- |
| IP-MS | 7 | Ovod, V., Ramsey, K. N., Mawuenyega, K. G., Bollinger, J. G., Hicks, T., Schneider, T., Sullivan, M., Paumier, K., Holtzman, D. M., Morris, J. C., Benzinger, T., Fagan, A. M., Patterson, B. W., Bateman, R. J., 2017. Amyloid β concentrations and stable isotope labeling kinetics of human plasma specific to central nervous system amyloidosis. *Alzheimer’s Dement.* 13, 841–849. |
| IP-MS | 17 | Nakamura, A., Kaneko, N., Villemagne, V. L., Kato, T., Doecke, J., Doré, V., Fowler, C., Li, Q. X., Martins, R., et al., 2018. High performance plasma amyloid-β biomarkers for Alzheimer's disease. *Nature*. 554, 249–254. |
| IP-MS | 18 | Schindler, S. E., Bollinger, J. G., Ovod, V., Mawuenyega, K. G., Li, Y., Gordon, B. A., Holtzman, D. M., et al., 2019. High-precision plasma β-amyloid 42/40 predicts current and future brain amyloidosis. *Neurology*. 93, 1647–1659. |
| IP-MS | 21 | Kaneko, N., Nakamura, A., Washimi, Y., Kato, T., Sakurai, T., Arahata, Y., Bundo, M., Takeda, et al., 2014. Novel plasma biomarker surrogating cerebral amyloid deposition. *Pro Jpn Acad Ser B Phys Biol Sci*. 90(9), 353–364. |
| IP-MS | 29 | Tosun, D., Veitch, D., Aisen, P., Jack, C. R., Jr, Jagust, W. J., Petersen, R. C., Saykin, A. J., Bollinger, J., Ovod, V., et al., 2021. Detection of β-amyloid positivity in Alzheimer's Disease Neuroimaging Initiative participants with demographics, cognition, MRI and plasma biomarkers. *Brain Commun.* 3(2), fcab008. |
| IP-MS | 30 | Schindler, S., Karikari, T., Ashton, N., Henson, R., Yarasheski, K., West, T., et al., 2022. Effect of race on prediction of brain amyloidosis by plasma Aβ42/Aβ40, phosphorylated tau, and neurofilament light. *Neurology*. 10.1212/WNL.0000000000200358. |
| IP-MS | 31 | Li, Y., Schindler, S. E., Bollinger, J. G., Ovod, V., Mawuenyega, K. G., Weiner, M. W., Shaw, L. M., Masters, C. L., Fowler, C. J., Trojanowski, J. Q., Korecka, M., Martins, R. N., et al., 2022. Validation of plasma amyloid-β 42/40 for detecting Alzheimer disease amyloid plaques. *Neurology*. 98(7), e688-e699. |
| IP-MS | 32 | West, T., Kirmess, K. M., Meyer, M. R., Holubasch, M. S., Knapik, S. S., Hu, Y., Contois, J. H., Jackson, et al., 2021. A blood-based diagnostic test incorporating plasma Aβ42/40 ratio, ApoE proteotype, and age accurately identifies brain amyloid status: findings from a multi cohort validity analysis. *Mol Neurodegener.* 16(1), 30. |
| IP-MS | 33 | Hu, Y., Kirmess, K. M., Meyer, M. R., Rabinovici, G. D., Gatsonis, C., Siegel, B. A., Whitmer, R. A., Apgar, C., et al., 2022. Assessment of a plasma amyloid probability score to estimate amyloid positron emission tomography findings among adults with cognitive impairment. *JAMA Netw Open*. 5(4), e228392. |
| IP-free LC-MS | 34 | Janelidze, S., Palmqvist, S., Leuzy, A., Stomrud, E., Verberk, I., Zetterberg, H., Ashton, N. J., Pesini, et al., 2022. Detecting amyloid positivity in early Alzheimer's disease using combinations of plasma Aβ42/Aβ40 and p-tau. *Alzheimer's Dement.* 18(2), 283–293. |
| Chemiluminescence | 38 | Yamashita, K., Miura, M., Watanabe, S., Ishiki, K., Arimatsu, Y., Kawahira, J., Kubo, T., Sasaki, K., et al., 2022. Fully automated and highly specific plasma β-amyloid immunoassays predict β-amyloid status defined by amyloid positron emission tomography with high accuracy. *Alzheimer's Res Ther*. 14(1), 86. |
| Chemiluminescence | 39 | Palmqvist, S., Janelidze, S., Stomrud, E., Zetterberg, H., Karl, J., Zink, K., Bittner, T., Mattsson, N., et al., 2019. Performance of fully automated plasma assays as screening tests for Alzheimer disease-related β-amyloid status. *JAMA Neurol*. 76, 1060–1069. |
| Chemiluminescence | 40 | Palmqvist, S., Stomrud, E., Cullen, N., Janelidze, S., Manuilova, E., Jethwa, A., Bittner, T., Eichenlaub, U., et al., 2022. An accurate fully automated panel of plasma biomarkers for Alzheimer's disease. *Alzheimer's Dement*. Advance online publication. https://doi.org/10.1002/alz.12751 |
| SIMOA | 20 | Verberk, I., Slot, R. E., Verfaillie, S., Heijst, H., Prins, N. D., van Berckel, B., Scheltens, P., Teunissen, C. E., van der Flier, W. M., 2018. Plasma amyloid as prescreener for the earliest Alzheimer pathological changes. *Ann Neurol*. 84, 648–658. |
| SIMOA | 35 | Vergallo, A., Mégret, L., Lista, S., Cavedo, E., Zetterberg, H., Blennow, K., Vanmechelen, E., De Vos, A., et al., 2019. Plasma amyloid β 40/42 ratio predicts cerebral amyloidosis in cognitively normal individuals at risk for Alzheimer's disease. *Alzheimer's Dement.* 15(6), 764–775. |
| SIMOA | 37 | Tanaka, T., Ruifen, J. C., Nai, Y. H., Tan, C. H., Lim, C., Zhang, Y., Stephenson, M. C., Hilal, S., Saridin, F. N., et al., 2021. Head-to-head comparison of amplified plasmonic exosome Aβ42 platform and single-molecule array immunoassay in a memory clinic cohort. *Eur J Neurol*. 28(5), 1479–1489. |
| ELISA | 25 | Pérez-Grijalba, V., Arbizu, J., Romero, J., Prieto, E., Pesini, P., Sarasa, L., Guillen, F., Monleón, I., et al., 2019. Plasma Aβ42/40 ratio alone or combined with FDG-PET can accurately predict amyloid-PET positivity: a cross-sectional analysis from the AB255 Study. *Alzheimer's Res Ther.* 11(1), 96. |
| Multiple | 22 | Janelidze, S., Teunissen, C. E., Zetterberg, H., Allué, J. A., Sarasa, L., Eichenlaub, U., Bittner, T., et al., 2021. Head-to-head comparison of 8 plasma amyloid-β 42/40 assays in Alzheimer disease. *JAMA Neurol*. 78, 1375–1382. |
| Multiple | 28 | Zicha, S., Bateman, R. J., Shaw, L. M., Zetterberg, H., et al., 2022. Comparative analytical performance of multiple plasma Aβ42 and Aβ40 assays and their ability to predict positron emission tomography amyloid positivity. *Alzheimer’s Dement*. 1-11. |
| Multiple | 24 | Keshavan, A., Pannee, J., Karikari, T. K., Rodriguez, J. L., Ashton, N. J., Nicholas, J. M., Cash, D. M., et al., 2021. Population-based blood screening for preclinical Alzheimer's disease in a British birth cohort at age 70. *Brain.* 144(2), 434–449. |
| Multiple | 36 | De Meyer, S., Schaeverbeke, J. M., Verberk, I., Gille, B., De Schaepdryver, M., Luckett, E. S., Gabel, S., et al., 2020. Comparison of ELISA- and SIMOA-based quantification of plasma Aβ ratios for early detection of cerebral amyloidosis. *Alzheimer's Res Ther*. 12(1), 162. |

eFigure 1. Diagram of Literature Search


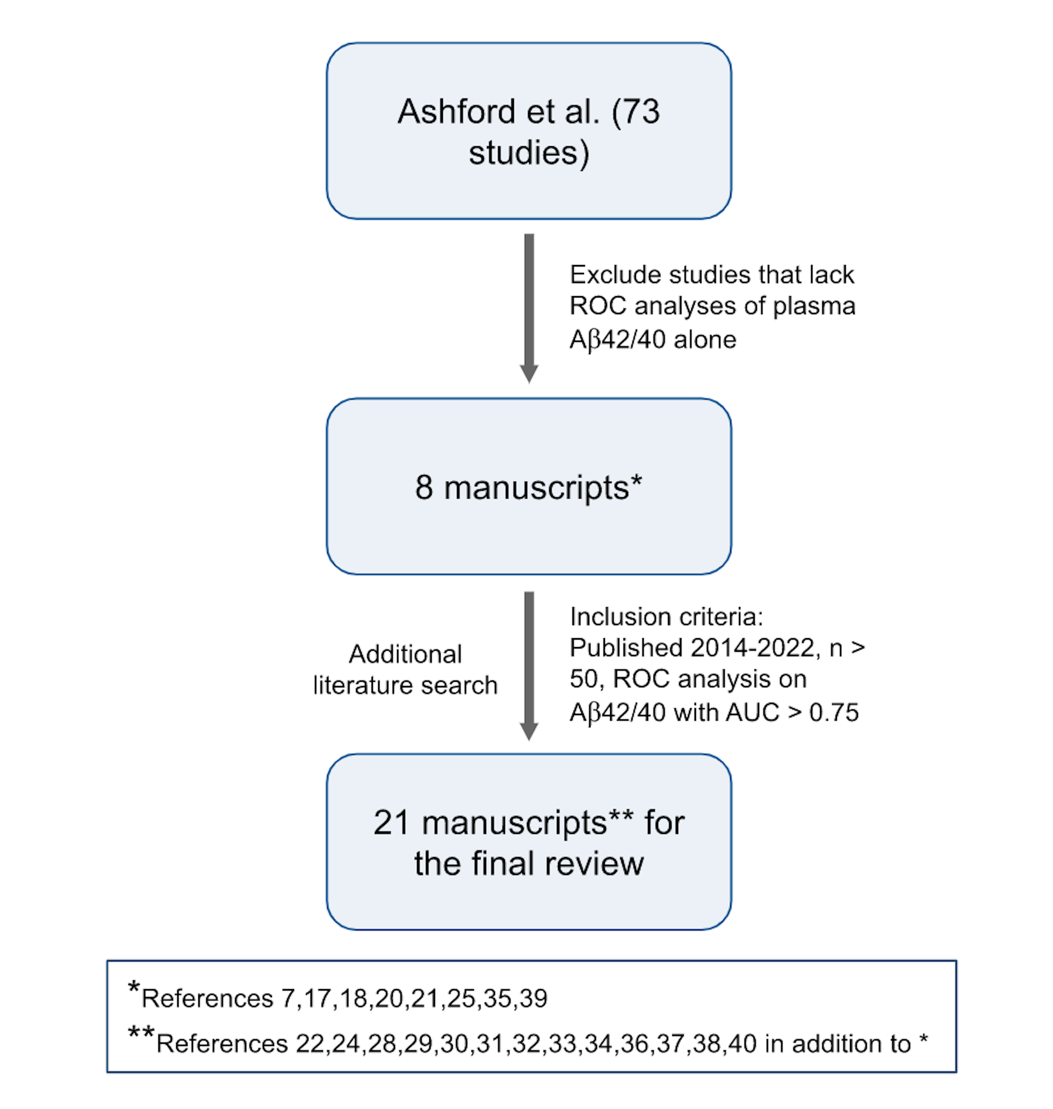

Supplement: Supplementary file 1 — Additional file 1: eTable 1. List of Identified Manuscripts to be Included in Review includes all citations, reference numbers, and types of assays studied for manuscripts included in the review. eFigure 1. Diagram of Literature Search is a schematic depicting how manuscripts were found for inclusion in this review, including a list of references at each step. [file 13195_2022_1117_MOESM1_ESM.docx]
